# Interpretable and Predictive Deep Neural Network Modeling of the SARS-CoV-2 Spike Protein Sequence to Predict COVID-19 Disease Severity

**DOI:** 10.3390/biology11121786

**Published:** 2022-12-08

**Authors:** Bahrad A. Sokhansanj, Zhengqiao Zhao, Gail L. Rosen

**Affiliations:** Ecological and Evolutionary Signal-Processing and Informatics Laboratory, Department of Electrical & Computer Engineering, College of Engineering, Drexel University, Philadelphia, PA 19104, USA

**Keywords:** COVID-19, SARS-CoV-2, coronavirus, deep learning, neural networks, machine learning, explainable artificial intelligence, computational biology, bioinformatics, viral genomics

## Abstract

**Simple Summary:**

As COVID-19 shifts from pandemic to endemic, emerging variants may be more or less virulent. Predicting whether an emerging COVID-19 variant has of high risk of causing severe disease is needed to plan for potential burdens on hospital capacity and protecting vulnerable populations. However, it takes time to do laboratory and animal experiments to determine whether a new genetic variant might be more severe, and the results may not be representative of when the virus infects humans. By the time there is epidemiological data on the severity of disease associated with a new variant, it can be too late for designing an optimal public health response. There is a critical need for computer models that can predict severe disease risk from genetic sequence data, which can be obtained from just the first few infections in a potential incoming wave. Two key challenges make computer modeling difficult: (1) sequence changes are complex, and (2) using historical data to predict future disease requires accounting for the confounding effects of changing patient demographics, improving therapeutics, and increased vaccination. In this paper, we introduce a novel interpretable deep learning architecture to solve this problem, demonstrating that it can make robust predictions for emerging variants.

**Abstract:**

Through the COVID-19 pandemic, SARS-CoV-2 has gained and lost multiple mutations in novel or unexpected combinations. Predicting how complex mutations affect COVID-19 disease severity is critical in planning public health responses as the virus continues to evolve. This paper presents a novel computational framework to complement conventional lineage classification and applies it to predict the severe disease potential of viral genetic variation. The transformer-based neural network model architecture has additional layers that provide sample embeddings and sequence-wide attention for interpretation and visualization. First, training a model to predict SARS-CoV-2 taxonomy validates the architecture’s interpretability. Second, an interpretable predictive model of disease severity is trained on spike protein sequence and patient metadata from GISAID. Confounding effects of changing patient demographics, increasing vaccination rates, and improving treatment over time are addressed by including demographics and case date as independent input to the neural network model. The resulting model can be interpreted to identify potentially significant virus mutations and proves to be a robust predctive tool. Although trained on sequence data obtained entirely before the availability of empirical data for Omicron, the model can predict the Omicron’s reduced risk of severe disease, in accord with epidemiological and experimental data.

## 1. Introduction

During the COVID-19 pandemic, an unprecedented amount of sequence data has been generated for SARS-CoV-2, the novel coronavirus that causes COVID-19. As of October 2021, when the data for most of the studies in this paper was collected, nearly 4 million sequences are available to researchers from the GISAID initiative (http://www.gisaid.org, accessed on 14 November 2022) [1]. As of 22 December 2021, that number had grown to nearly 6.4 million sequences. There is a critical need to translate abundant biological sequence data to as much biological information as possible. This goal is particularly urgent as the virus continues to rapidly mutate and change, often in fundamental ways that affect disease severity. In particular, as COVID-19 shifts from a pandemic to an endemic state, emerging genetic variants of the virus may have different health burdens and require different public health responses [2]. As a result, there is a critical need for modeling methods capable of predicting the risk of severe disease burden on hospitals and vulnerable populations as a result of continued viral evolution [3].

This paper focuses on mutations within the spike protein of SARS-CoV-2 in particular, because of its crucial role in COVID-19 infection and the immune response [4,5,6]. The spike protein is the key target for COVID-19 vaccines and antibody therapies [7,8,9]. The spike protein is responsible for cell membrane entry in all coronavirus species. Generally, the spike proteins of coronaviruses have two subunits, S1, which is responsible for binding to the receptor, and S2, for membrane fusion [10].

As the COVID-19 pandemic has continued, the SARS-CoV-2 spike protein has accumulated mutations, and variant protein sequences have emerged [11]. While coronavirus mutations are most commonly substitutions, although there have been frequent insertions and deletions as well [12]. Even insertions from other viral genomes have been observed, such as a potential insertion from human RNA [13]. Early on in the pandemic, spike protein sequence variants began to be observed, such as a substitution D614G, which rapidly became dominant as the pandemic began to spread within Europe [14,15]. Since then, as SARS-CoV-2 has been exposed to hosts around the world, a substantial amount of mutation has occurred [16]. SARS-CoV-2 variants can have critical differences in the level of virulence, pathogenicity, immune evasion (with implications for therapeutic antibodies and vaccine efficacy), and transmissibility [17].

Initial work on the impact of spike protein variants focused on the D614G substitution in the spike protein, which was found in an animal model to potentially increase in fitness and transmissibility [18]. However, as other studies accumulated, it proved difficult to show a clear impact on virulence or transmissibility, although clinical samples with the variant were found to have higher virus titers [19]. The wide spread of the Alpha (B.1.1.7) variant starting in December 2020 did suggest that it had an increase in transmissibility over wild type and previous variants [20], although it is unclear that it resulted in more severe disease [21,22,23,24]. Epidemiological estimates have shown that the Delta (B.1.617.2) variant has increased transmissibility [25]. Moreover, laboratory studies of Delta show increased fitness over previous variants, in particular due to enhanced viral replication as a result of a modified furin cleavage site of the spike protein [26,27].

The complexity of genetic changes within the SARS-CoV-2 spike protein suggest that a large database of viral genomes from a diverse population of patients is necessary to infer whether genetic changes can impact disease severity. Fortuitously, within the more than five million sequences uploaded to GISAID, a small yet substantial fraction (nearly 220,000 of over 5,500,000 sequences as of 27 November 2021) have some metadata about the clinical status of patients. Such metadata can include whether the sample comes from someone who is alive or dead, whether they were in the ICU or had mild or severe disease, etc. Generally, only one piece of information (such as “alive” or “dead,” or “mild” or “severe”) is conveyed through the metadata annotation. The GISAID dataset and focused patient studies (which are often the source of the data available on GISAID) have enabled researchers to study the potential link between sequence variation and virulence.

Given the availability of patient metadata information for at least a subset of GISAID data, there is an opportunity to create a model that links sequence variation to the risk of severe disease. However, approaches to this problem thus far have run into problems of inconsistent trends in data and having to address significant confounding by, for example, (i) factors which increase disease risk such as patient age, sex, and preexisting conditions, and (ii) increasing rates of vaccination and improved therapeutic techniques over time. In particular, the approaches that previous studies have considered at looking at the relationship between viral genome or spike protein changes and disease severity have included genetic comparison [28,29,30], meta-analysis of published clinical studies [31], and analysis of mutations in GISAID samples with clinical metadata [32]. These studies were able to find some correlation of symptomatic or severe disease with spike variants such as D614G, although they were not verified elsewhere. Another analysis of country-based case fatality rates (CFR) found either no relationship between mortality and clade, at least through March 2021 [33]. To the extent that patient status in GISAID metadata does show variation, other studies using logistic regression and conventional statistical techniques found it to largely reflect worse outcomes for older males, consistent with commonly understood clinical experience, while it was difficult to discern variation at the clade or sequence levels [34,35].

However, over time even well-performing classifiers began to perform worse than they did before in updated studies, in particular after Omicron began to emerge [36]. One approach did find a mutational signature using random forests based on mild/severe disease classifications, but the mutations it found like V1176F and L5F in the Spike gene have not been validated [37]. Other more complex analysis has included the use of deep neural networks, in particular, a combination of convolutional neural network (CNN) and recurrent neural network (RNN), which attempted to predict what mutations would increase virulence severity based on countrywide death statistics [38]. Another approach that has been developed and recently validated is the use of Bayesian multinomial logistic regression to infer growth rate from individual mutations; from that, the authors posited that the mutations correlated with high growth rate also conferred fitness and transmissibility benefits [39]. Another approach has been to use random forests to predict the impact of both known and “virtual” mutations on the structural biology of the virus and in turn impact on affinity to neutralizing antibodies and transmissibility [40]. Another group has applied “ProtBERT” protein sequence embeddings obtained through self-supervised learning with BERT to SARS-CoV-2 in combination with *k*-means clustering to identify groups of correlated mutations [41]. Another work has combined models of cell receptor binding and immune epitope alteration with transformer-based deep learning models to predict the fitness advantage of SARS-CoV-2 mutations [42]. Our group has also recently explored the potential of mixed effects gradient boosting models to predict severity in view of confounding variables [43]. Notably, even though complex sequence changes can be analyzed using machine learning techniques, an important problem is how to interpret the reasons for the predictions, particularly in the presence of confounders such as patient demographics and vaccination.

In this paper, we propose to address the problems of complex sequence mutations, confounding, and model interpretation through a deep learning architecture that relies on “attention” neural network mechanisms [44,45], which have been applied to interpret deep learning models for text classification by highlighting positions in text based on attention values [46,47,48]. Deep learning models combining CNN with attention have been used to identify sequence motifs for functional genomics, e.g., transcription factor binding site detection [49,50]. Our group has shown that attention in combination with a LSTM-based sequence model can be used to obtain insight into taxonomic and phenotypic classification of 16s ribosomal RNA sequences of bacteria [51], as well as gene ontology classifications of protein sequences [52]. Recently, transformer-based architectures, which are built on multiple attention modules, have emerged as being important in NLP. The attention within transformers has been proposed as a source of explainability. For example one work demonstrated how different attention heads attended to different aspects of a learning task to identify nucleotide motifs for promoter sequences [53]. The deep learning architecture presented in this paper advances previous work by incorporating multiple interpretative layers and also including inputs from demographic variables and, specifically, sample collection date, as a way to account for the confounding effect of patient characterstics and increased vaccination rates over time.

This paper presents results of learning and testing the proposed Transformer-based architecture. First, a model is developed by regression of sequence data to the date of a sequence’s first emergence, as a way to qualitatively validate the significance of the embedding and attention-based interpretations generated by the model. Second, a model for predicting disease severity of sequences is developed by using patient metadata available for a subset of sequences in the GISAID databases and using attention to identify mutations that may be related to heightened risks of severe disease. Model predictions are validated on the Omicron variant, which was first reported on 25 November 2021 from sequences in Southern Africa and then rapidly grew and became the dominant variant worldwide [54]. Predictions on Omicron are made based on sequence data in GISAID available only up to October 2021, thereby excluding any Omicron sequences. Although the Omicron variant has different sequence properties from other variants found before its emergence, the results described in the final section of the paper demonstrate that the model may be useful in making computational predictions before any empirical data emerges, specifically by predicting the relative risk of disease severity as compared to Delta and Alpha. This proof of concept shows the potential for a predictive computational model that can provide a starting point for epidemiological guidance before health observations and laboratory data are collected.

## 2. Materials and Methods

### 2.1. Data Collection and Pre-Processing

As described further in the Results section, the studies shown in this paper employ two sets of SARS-CoV-2 spike protein sequences: a full set of SARS-CoV-2 spike protein sequences submitted to the GISAID database [1], as well as a subset of protein sequences which were provided to GISAID with patient metadata information specifically, and protein sequences from coronavirus of all genera. The procedures for collecting and processing sequence data are outlined for each type below in turn.

#### 2.1.1. SARS-CoV-2 Spike Protein Sequences

A FASTA file of protein sequences was downloaded from the GISAID database [1], http://www.gisaid.org, accessed on 14 November 2022. The accession identifiers of the GISAID sequences and metadata used in this study are collectively provided by the GISAID EPI-SET identifier EPI_SET_20220105ms, and are thereby available from GISAID subject to GISAID’s terms and conditions. The acknowledgments and sequence identifiers can be accessed at https://epicov.org/epi3/epi_set/EPI_SET_20220105ms, accessed on 14 November 2022. The spike protein sequences are preprocessed by GISAID by multiple sequencing alignment, identifying open reading frames, and translating nucleotide sequences to obtain protein FASTA files. The FASTA file is then parsed to obtain a file with only the Spike protein sequences. (GISAID also offers a FASTA file of only Spike protein sequences; however, that was not used for this paper.) Unless indicated otherwise, the protein sequences used in this paper are those submitted and preprocessed by GISAID as of 16 October 2021. The metadata annotation file from GISAID including sequences collected and processed as of 1 October 2021 was also downloaded from the GISAID website. This metadata file included the collection date, annotated Pangolin lineage [55], Nextstrain-identified clade, and geographical information.

This paper refers to “raw” and “aligned” sequences. Raw sequences are exactly those that were contained in the FASTA file downloaded from GISAID. Aligned sequences are generated using the local pairwise Striped Smith-Waterman (SSW) method [56], with BLOSUM62, in the scikit-bio package in Python 3.8 [57]. Following alignment, the resulting protein sequence has all insertions and deletions positioned with respect to the consensus Spike reference sequence (Wuhan-Hu-1 isolate) which was obtained by multiple sequence alignment of early genome sequences [58]. Variant single site polymorphisms can then be identified in accord with convention. First, the sites in the aligned data sequence corresponding to a gap in the aligned reference sequence were shifted such that any insertion was relative to the reference. Aligned sequences are then padded at the beginning and end of the aligned region (if it was less than the 1273 residue length of the reference sequence) with a mask character “*” to formulate a 1273-residue long sequence. While not generally done for the studies presented in this paper, it is also possible to filter aligned sequence data by removing any sequences containing any “*” or X (ambiguous amino acid) characters. The source code for the alignment and processing of aligned sequences is provided at https://github.com/EESI/Covid, accessed on 14 November 2022.

#### 2.1.2. GISAID Sequences Containing Patient (Clinical) Status Metadata

The analysis in this paper considers the subset of sequences that were available and aligned by GISAID to identify spike protein sequences as of 1 October 2021 for which patient metadata (a “patient status” entry) were then available on GISAID (155,545 records in total). Each record is for a SARS-CoV-2 sequence from a distinct, individual patient. All records further include additional metadata: host, continent/country/region of collection, Pango nomenclature lineage, NextStrain clade, sample collection and submission date, patient age, and patient gender. Based on the “host” metadata field, all non-human samples are removed from the dataset.

Patient metadata uses non-standardized text that may include misspelling and terms in different languages. For the analysis presented here, metadata entries are first assigned to a “Status”, which translates entries that are different spellings or synonyms for the patient status. Supplementary Table S1 shows all of the unique metadata entries and corresponding “Status” designation in the full patient metadata set along with the corresponding “Status” designation. The resulting status is then assigned to a “Category”, following common usage and categories defined by the United States National Institutes for Health (NIH) COVID-19 guidelines [59]. For example, ICU admission or mechanical ventilation are categorized as “Severe”. Metadata entries are categorized as “Unknown” if they are (i) entered as “unknown” (or other synonym); (ii) cannot be interpreted or are errors, e.g., in some cases patient age or sample location appear to have been entered as patient status; or (iii) cannot be deciphered in terms of case severity, e.g., “recovered”, “alive” or “symptomatic” patients may have had either mild or severe disease. By contrast, “released” patients are assumed to have been previously hospitalized, and are thus are classified similarly to (presumably currently) hospitalized cases. The categories are then assigned to “Mild”, “Severe”, and “Unknown” classes, according to the NIH categories where there is sufficient information. Table 1 shows the categories and the status designations, obtained from metadata entires according to Supplemental Table S1, included in each Category.

As further detailed in the paper, “age”, “gender”, and “date” metadata are also included in models. While the term “gender” is used in this paper to align with the GISAID metadata definition, the entries refer to sex and gender interchangeably. In addition, manual review is used to ensure that all“gender” entries decipherable as male or female are included in the data, which requires interpreting misspellings and foreign language words such as “Homme” (French for “man”). Remaining entries are classified as “Unknown”. Patient ages are assigned to an integer age where such an age may be deciphered from the “age” metadata field. In some cases, “age” is provided as a range, e.g., “21–30”, in which case it is replaced with the mid-point, e.g., 25. In some cases, patient age was entered as unknown or is not decipherable, and it is then dropped from the data. Date in this study refers to the sample collection date noted by GISAID. In some cases, the date appears to have been erroneously annotated; for example, in some cases the date is from before the onset of the pandemic, or is otherwise missing or indecipherable. Such samples are removed from the data. Integer dates in this study have been normalized to the original sample collection dates by setting Day 0 to 1 December 2019, which is before the first sequenced samples from COVID-19 patients were collected. To conserve limited training data, samples with incomplete dates, i.e., missing date or month, are retained and assigned to the latest possible date, e.g., “2021-01” would be annotated as “2021-01-31”. To demonstrate the distribution of patient demographic metadata for the principal (most prevalent) Pangolin lineage classifications, Table 2 shows the percentage of severe cases, mean age, and percentage of male patients for the patient metadata validation set descibed above.

### 2.2. Model Architecture

**Figure 1:**
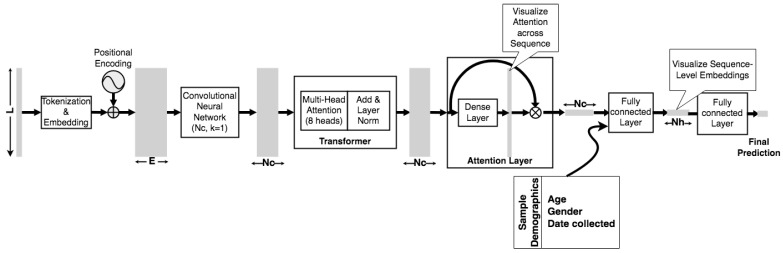
Deep learning architecture used in this paper. The input is a sequence of length *L*, tokenized as described in the Methods section. The input is embedded using trainable weights in a matrix with both token and positional information. The embedding is compressed using a CNN layer with *N_c_* filters and fed to a multi-head attention Transformer encoder. The encoder output is processed by a self-attention layer, enabling attention visualization corresponding to each amino acid site in the protein sequence. The output of the attention layer is then optionally concatenated with an additional vector of integers, here additional patient characteristics such as age. The result is input to a densely connected layer with *N_H_* nodes, where, typically, *N_H_* << *L*, allowing us to analyze the distances between sample vectors embedded in an optimal classification space, for example in a t-SNE plot of embedded vectors. Finally, a sigmoid activation node can provide a binary class prediction.

Figure 1 shows an overview of the deep neural network model architecture. In this paper, the input is a protein sequence of length 1273. Protein sequences are tokenized by individual residue or other character. Each amino acid (residue) and the deletion symbol “-” is represented by a distinct nonzero integer. A position with the symbol “*”, which was padded as part of the alignment process described above, is assigned to zero, as are ambiguous amino acids (i.e., amino acids that cannot be determined from sequencing because the sequencing data includes ambiguous bases) with symbols X, B, J, or Z. The sequence of tokens is then transformed into a matrix of embeddings of each token in the input sequence, in which the embedding includes both token and positional information. The embedding begins with a random initialization, and its parameters are then allowed to learn during the learning task.

A transformer architecture is used for sequence encoding, as first described in developing an encoder-decoder architecture for machine translation of text [60]. The transformer architecture is a modular multi-head structure, in which each head consists of an attention layer and feed forward neural network, wherein the head outputs are added and normalized to provide a sequence encoding [60]. The token and position embedding dimension (*N_E_*) that are used in this paper are large, because the sequence lengths is 1273 and the embedding for transformer-based architectures includes positional as well as token information. While certain architectures or parallel methods can train Transformer-based models with such long sequences, this sequence length and the potentially longer sequence length of other viral proteins motivates methods for more efficient processing. Consequently, the architecture presented here includes an *optional* convolutional neural network (CNN) layer with a kernel width of 1 and *N_C_* filters before the transformer is added as a way to reduce the required size of the transformer heads in order to allow us to load the model on a single processing unit for efficient computation. The optional CNN layer can reduce the effective size of the Transformer, which allows for less memory usage, larger batch sizes, and faster training times per epoch, however, at the cost of losing sequential information because a CNN can only maintain limited local information.

Encoded sequences are then used to do classification or regression, depending on the learning task. Two layers are added to assist in classification but also allow us to visualize, and potentially interpret trained models. The first is a self-attention layer following the structure inspired by [48] and which is utilized in [51], where it was combined with a Bidirectional Long Short Term Memory (Bi-LSTM) encoder. Similarly, in this paper, although it is not used with a Bi-LSTM, the structure is applied to readily access sequence-level classification, since the attention heads cannot individually, nor in sum, provide full attention visualization across the sequence (see, e.g., [61]). The second is an intermediate *N_H_*-dimension densely connected layer, which can project an *N_H_*-dimensional embedding of the sequence as a whole. In the studies shown here of patient status metadata classification, the integer-valued demographic variables are concatenated with the *L*-dimensional (sequence length) Transformer output and fed to the *N_H_*-dimensional embedding layer. As explained above, integers are used for age and sample collection date, and a 0/1 value for gender. These embeddings are then fed to an output layer. The output layer can be either of the following: (1) a single node with sigmoid activation for either binary patient clinical status classification or the regression used to predict the Omicron variant’s reduction in neutralizing antibody activity, or, (2) a clipped linear output restricted between 0 and 1 for regression to sample date.

Model analysis and interpretation can occur on two distinct scales c: First, *attention*, which shows where the model attends along the sequence (i.e., amino acid position in a protein sequence) and can identify patterns at the sequence level [51,62]. Second, *embeddings*, in which the *N_H_*-dimensional encoding of a sequence at the layer shown in Figure 1 (see above) is obtained for a trained model, and then the embeddings of a group of sequences post-training are visualized or clustered to find patterns at the group level [63]. The authors have previously shown that, for example, embeddings can be plotted to reveal underlying taxonomic categorization in the context of microbial 16s rRNA sequences used to predict inflammatory bowel disease status [51].

### 2.3. Implementation

#### 2.3.1. Model Parameters

For the model as shown in Figure 1, the embedding dimension *N_E_* is set to 1500, the CNN has *N_C_* of 300 filters, the Transformer block contains 8 attention heads, a feedforward network (FFN) of 64 dimensions, with dropout of 0.1; *N_H_* is 64, and no other dropout is added. To obtain an optimal combination of hyperparameters, cross-validation was performed on a random partition of 50% of the training data set used in the patient metadata study, i.e., samples with patient metadata found at least five times in the GISAID database prior to 12 September 2021. Hyperparameters were varied as follows: number of Transformer heads between 4, 6, and 8; number of FFN nodes between 32, 64, and 96 nodes; transformer dropout between 0.0, 0.1, and 0.2; dropout of 0.0, 0.1, and 0.2 after the embedding layer; *N_C_* at 300, 400, and 500; *N_H_* at 64 or 128; and *N_E_* of 1000, 1200, 1500, and 1800.

The number of epochs for training based is selected based on the observation that, in general, the number of epochs required for classification tasks is much lower than that for regression tasks. In the case of binary classification for patient metadata, the model is trained for 75 epochs, after evaluating epoch ranges from 50 to 200. Early stopping is not used with a distinct validation set, as that was found that reduces the training data and causes underfitting, and instead early stopping is set at 20 epochs with no net change in the binary cross-entropy loss in training data taken as a whole. For the regression to sample date task, training is limited to 600 epochs, with an early stopping time set at 200 epochs. Multiple training runs were performed to evaluate run-to-run consistency given the randomization of initial weights and other inherent randomization of training.

As further described in the Results section, the transformer-based model was benchmarked against eXtreme Gradient Boosting (XGBoost), a decision tree-based ensemble learning method [64]. In the case of XGBoost, one-hot encoding is used to represent all the sequence variants found in the training data. For XGBoost, after hypertuning, the following parameter values were used for XGBoost: subsample rate of 0.8, lambda regularization of 1.0, maximum depth of 10, learning rate of 0.001, gamma of 0.0, column sample by tree of 0.8, and the GPU-optimized predictor. XGBoost is implemented using the Python package in xgboost 1.5.1 in the Google Colab GPU runtime environment. Because XGBoost results may also change due to randomization, the run-to-run consistency of XBoost is evaluated under different random number generator seeds. The results shown in this paper were found to be consistent with negligible standard deviation between runs on the same data and with the same parameters.

#### 2.3.2. Training and Validation

Model training was done using the standard Adam gradient descent fitting algorithm in Tensorflow 2, with binary cross-entropy and mean squared error as the loss functions for classification and regression tasks respectively. The learning rate parameter is set to $1\times 10^{-4}$, after evaluating rates of $5\times 10^{-5}$, $1\times 10^{-5}$, $1\times 10^{-3}$, $5\times 10^{-4}$, $1\times 10^{-2}$, and $1\times 10^{-1}$ within the hyperparameter search data set described above. In this paper, the model is trained 75 epochs, after evaluating epoch ranges from 25 to 200. Early stopping is employed, though not with a subset of the training data set to avoid reducing the resulting training data set size and thereby risking underfitting. Instead, early stopping is set at 20 epochs with no net change in the binary cross-entropy loss. Multiple training runs are used to evaluate run-to-run consistency given the randomization of initial weights and other inherent randomization of training. Where not shown, standard deviations across runs were not found to be greater than reported precision.

## 3. Results

### 3.1. Regression Based on Sample Collection Date

The interpretability of the model architecture through visualizing embeddings and attention can be validated by training models on an exemplary machine learning task. Shown here are the results of regression to the sample collection date of sequences in a database of raw, unprocessed spike protein sequences available on GISAID as of 1 October 2021. Specfically, the the earliest sample collection date included in the GISAID database is predicted for each distinct sequence in the data set. The training data set consists of sequences that are found more than four times in the database. The validation set consists of sequences found only two or three times in the database. Notably, some sequences are found very frequently, such as certain sequences in the B.1.1.7 (Alpha) and B.1.617.2 (Delta) lineages. For the purpose of this task, these sequences are included once and weighted equally to other sequences. Trivially, the regression should “predict” that a sequence will have appeared at the same time as others within the same lineages, as the lineages are defined based on sequence similarities at particular sites with the SARS-CoV-2 genome. In particular, the lineages, following the Pango lineage schema [55] are based in large part on changes in the spike protein sequence, which should be reflected in the model, which is trained on spike protein sequence data. Accordingly, trends in the embedding and attention would be expected to reflect the classification.

As an initial matter, the trained model’s prediction error is within a reasonable error margin. To do so, the square root of the mean squared prediction error (RMSE) on validation set data is normalized by the RMSE of simply predicting each label with the overall mean value of all labels. The RMSE of the trained model was 0.0962 and normalized RMSE was 0.534. By comparison, the normalized RMSE on the training data set is 0.37. Therefore, while RMSE increases when generalizing the model to the validation set, it remains significantly below 1.0, indicating that the model predicts significantly better than chance.

**Figure 2:**
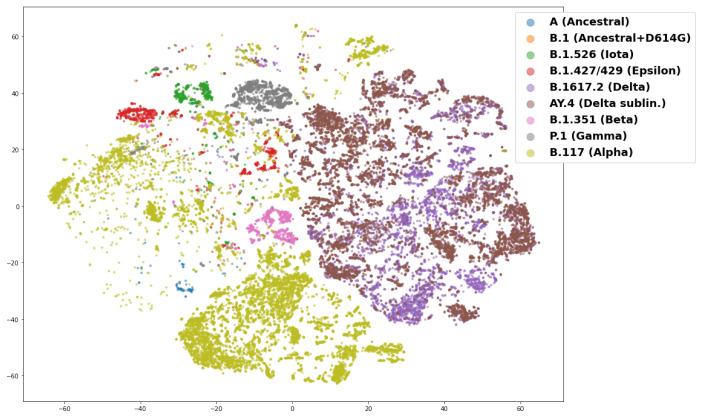
Plot of a two-component t-SNE analysis (with perplexity of 50) for the 64-dimensional embeddings of raw spike protein sequence data following a prediction task for the date of first sample collection (in days since 1 December 2019) of a particular sequence. The training data consisted of sequences found 4 or more times in the global data set (all spike protein sequences available on GISAID as of 1 October 2021), and the validation data shown here consists only of raw, unprocessed spike sequences found 2 or 3 times in the global data set. The lineages shown in this plot are provided in the legend and consist of the ancestral lineage (A), as well as variants of concern and interest including Alpha (B.1.1.7), Beta (B.1.35.1), Iota (B.1.526), Gamma (P.1), Epsilon (B.1.427/429) and Delta (B.1.617.2), and AY.4, a Delta sublineage. Many lineages can be clustered with the embeddings; Delta and the AY.4 Delta sublineages cluster into a large but well separated group, while Alpha is spread out.

Figure 2 further demonstrates the model’s performance by showing that a t-SNE (t-distributed stochastic neighbor embedding) plot [63] of the *N_H_*-dimensional (*N_H_* = 64) embeddings of sequence samples in the validation data set. Figure 2 is consistent with the intuition described above: A lineage will generally consist of sequences that emerge at a common point in time. As such, modeling sequences by the date of their first emergence groups the sequence embeddings from the same lineages together and separates them from embeddings of other lineages. Notably, these samples are from a validation data set which includes raw sequence data, i.e., they include ambiguous residue calls and are of all different sizes. Most sequences will have either 1273 amino acids (wild type) or a few less (i.e., given deletions), but 2619 sequences of the 58,747 sequences are of length less than 1270 and 634 are fragments with a length less than 500 amino acids. Moreover, a majority of sequences—39,008—include one or more ambiguous amino acid calls. These issues may account for instances where the sequence embeddings do not group by lineage as expected. The overlap between B.1.617.2 (Delta) and AY.4 is also expected, as AY.4 lineages (e.g., AY.4.2) are recognized as Delta sublineages which have emerged during the Delta outbreak [65]. In sum, Figure 2 shows that the model can learn SARS-CoV-2 lineages and predict the date that a sequence emerged (was first collected), without doing (1) further alignment, (2) phylogenetic tree-building, or (3) classification based on sequence polymorphisms.

**Figure 3:**
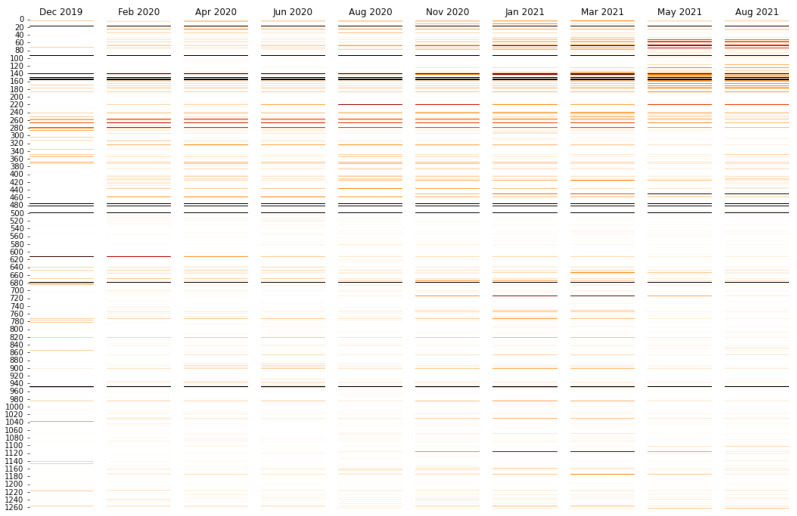
Mean attention at each amino acid position for sequences in the validation data set (aligned sequences occurring 2 or 3 times in the 1 October 2021 global data set) categorized into bins based on the date of first emergence. The intensity of the color is proportional to the attention log-normalized by the maximum attention for all sequence positions, such tat the peak values are black and intermediate attention levels are various shades of red. The mean attention values vary over time as waves of new virus genome variants emerge. For example, spike protein amino acid sites between 600 and 1200 become important as Alpha emerges in the winter of 2020-21. Some specific positions, e.g., 478 and 681, have high attention throughout time because of their changes from earlier in the development of the pandemic, Alpha, to the later Delta variant. More attention is paid to other sites, such as 452, which is characteristic in Delta, as the become a more significant indicator of the difference in a novel variant as it emerges.

**Figure 4:**
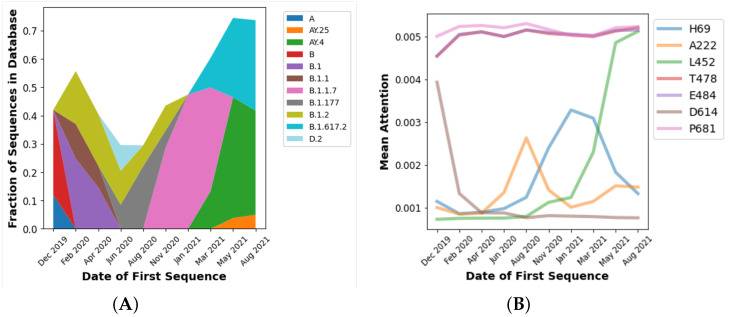
(**A**) Relative prevalence of the three most frequently observed lineages (by sequence) in the 1 October 2021 data set in each time period identified in Figure 3. Because less frequently observed lineages are excluded for clarity, the values sum to less than 100%. (**B**) Mean attention levels for exemplary protein sequence positions with date of first emergence in time periods corresponding to Figure 3. The legend shows the amino acid in the reference (wild type) sequence for each position.

Visualizing attention provides interpretation of the model. The interpretive potential of attention is demonstrated by the results of modeling the aligned data set, in which sequences were aligned to the reference sequence and padded with mask characters for an equal length of 1273 amino acids. This provides more consistent attention values across sequences in the validation data set which are of different lengths. There is no correction, however, for masking or noise symbols in the spike sequences. The prediction errors were in line with the raw sequence data set, with a RMSE of 0.0970 and normalized RMSE of 0.53. Figure 3 shows the mean attention for each sequence in the validation data set (sequences occurring more than 2 or 3 times in the global 1 October 2021 data set) binned by date of first emergence. Figure 3 shows that attention values change as the pandemic progresses, which corresponds with the introduction of new variations in the spike protein sequence over time. Figure 4A demonstrates how major lineages have emerged and retreated during the time periods corresponding to the binned dates in Figure 3.

Comparing Figure 3 and Figure 4B reveals that attention levels in the Winter of 2020 and Spring of 2021 at various positions increase relative to earlier time points, and in some cases decrease later in 2021. Broadly, the attention patterns correspond to the increase and decrease in prevalence of lineages in Figure 4A, in particular the B.1.1.7 (Alpha) wave, which succeeded earlier lineages that originated in China and then spread to Europe and North America, and which was followed by a wave of Delta (B.1.617.2) and sublineages. Figure 4B shows the patterns in greater detail for exemplary sequence positions. For example, a characteristic Delta mutation, L452R, increases as 2021 continues and Delta emerged [66]. By contrast, the attention at site 614 is highest earlier in the pandemic. This corresponds to D614G being the first major mutation to emerge after the pandemic began, and then subsequently becoming a ubiquitous aspect of all subsequent lineages [67]. Consistent with the rise and fall of the prevalence of B.1.1.7 shown in Figure 4A, Figure 4B shows the rise and fall of the attention level at position 69. Positions 69/70 are where deletions occur in the B.1.1.7 (Alpha) lineage, which have not been found in Delta and Delta sublineages which began to dominate later in 2021.

There is a more complicated picture for position 222, as the A222V mutation emerged late in 2021 in the Delta AY.4.2 sublineage [65]. The A222V mutation, however, had also been found in mid-2020, which corresponds to the earlier peak in Figure 4B [68]. The reason why some attention values at certain sites remain consistently high over time could be because there consistently has been some substantial level mutations at those sites throughout the pandemic. For example, the attention at positions 478 and 484 track each other at high levels and coincide on the same track on the plot. This may be explicable because position 478 is within the receptor binding domain (RBD), and it had a high level of mutations in summer of 2020 [69] continuing through later in 2021 when the T478K mutation emerged as a characteristic mutation of the Delta lineage [66]. Similarly E484Q has been a characteristic mutation in the Delta lineage, but E484K has been an aspect of other pre-Delta, such as Beta and some Alpha sublineages [70,71,72]. Similarly, P681H was found in the highly prevalent Alpha lineage [73], and P681R was found in the Kappa and then later Delta lineage [74]. Accordingly, it may be the case that a consistently high attention suggests that the model pays attention to the site because it was not a point of variation at early time points. The attention does vary though in subsequent time periods where it is found in some important lineages (such as Kappa and then Delta) but no other highly prevalent lineages (e.g., Alpha). The model thus needs to use that site to predict the emergence date of sequences across all time periods.

### 3.2. Predicting COVID-19 Patient Outcomes Using Machine Learning Models Based on Both Sequence and Demographic Information

As detailed in the Methods section, GISAID samples with patient metadata are used to train a model for the relationship between severity of COVID-19 disease and the spike protein sequence. Approximately 147,000 of the 3.9 million samples available on the October 2021 cutoff date for inclusion in this study include any patient metadata as well as readable spike protein sequence information. Many of the metadata entries are simply “unknown” or not applicable to patient status, e.g., include information about sample collection. Additionally, as described in the Methods and shown in Supplemental Table S1, many descriptions cannot be used together in a coherent classification task. For example, it is not possible to compare patients who are “alive” or “dead”, as the patients who are not listed as being dead would have a wide range of outcomes from asymptomatic to ICU admission. Accordingly, the only samples that could be used for training or testing are those with metadata that could be assigned to two broad categories, “Mild” (0) or “Severe” (1) following the scheme in Supplemental Table S1, based on the NIH Clinical Guidelines for COVID-19 [59]. After eliminating samples which cannot be classified as Mild or Severe, 65,916 samples (corresponding to individual patients) remain.

The training data set is made up of samples which were (1) made available in the GISAID database as of 12 September 2021 and (2) found at least five times overall in the data to that point. All remaining samples made available up to 1 October 2021 in GISAID make up an independent validation set. The validation set thus includes samples with sequences made available on GISAID after September 12 or not found at least five times before September 12. As a result, there is no exact sequence overlap between training and test samples. Notably, there is often a lag between the sample collection date and when it is submitted to GISAID, meaning that some samples in the validation may have been collected prior to later samples in the training data set. Training and validation data sets include 44,003 and 21,913 samples respectively. The data are biased to Severe cases overall. Training data include 19,502 Mild (44.3%) and 24,501 (55.7%) Severe patient samples; validation data include 8948 (40.8%) Mild and 12,965 (59.2%) Severe patient samples.

**Figure 5:**
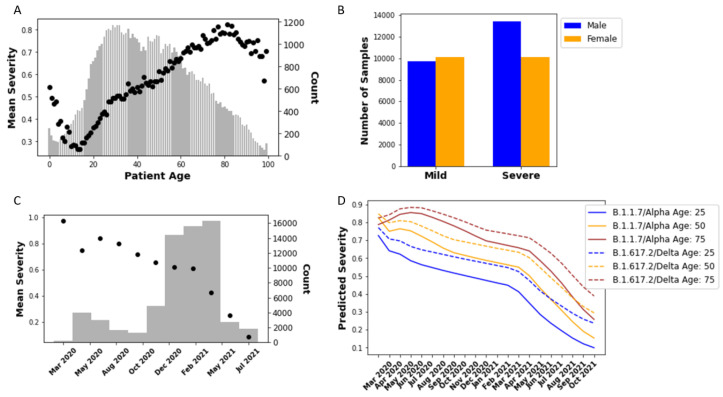
(**A**) Mean of clinical severity, ranging from 0 to 1 (Mild to Severe, respectively) (dots) and count of samples in GISAID database with available age metadata (bars) for varying patient age. (**B**) Number of mild and severe cases for each sex/gender as identified in the “gender” patient metadata field for GISAID samples. (**C**) Mean clinical severity varying by sample collection date; data are binned over time periods with the count shown by the bars. (**D**) Model predictions, including sequence, age, date, and gender information, of mean severity over time of patient samples with sequences from Alpha (B.1.1.7) and Delta (B.1.617.2) lineages. Three different ages are run, as indicated in the legend, and the gender variable is set to male. The predictions shown here are the averages for the 30 most prevalent sequences (frequency found in database) for each lineage. A prediction of variant severity can be made over time, accounting for time in pandemic and age of patient.

When developing a model to link sequence to disease outcomes, demographic variables must also be considered. Throughout the pandemic, there has been consistent epidemiological and clinical evidence that age and sex/gender (male) are major risk factors for more severe COVID-19 symptoms [75,76,77]. Earlier studies have shown that GISAID data also supported a correlation between the demographic metadata which are available—age and gender—to clinical severity [34,35]. Figure 5 shows the relationship between these variables and clinical severity, which show, as expected, a correlation with age (increasing age results in increasing mean severity) and sex/gender (male patients have a greater prevalence of severe outcomes). Notably, while between extremes of young and old there is a continual trend, at the extremes the trend diverges. Extreme age values, however, are represented by far fewer samples in the data set, and thus may be susceptible to study bias of which samples were sequenced. For example, if more infants (with sequenced samples in the GISAID database) were hospitalized, even for incidental reasons, that would be reflected as a more severe case. Or, if very old patients were sequenced as part of a study of elderly patients who had survived or had a milder course of disease than expected for their age, that would skew a small number of samples milder.

Besides patient demographics, the results show a substantial trend in the frequency of severe outcomes based on sample collection date. Figure 5C shows how the number of samples with severe disease in the GISAID database drops as a function of time through the pandemic. There has been a particularly sharp decrease since approximately February 2021. This trend has been consistent even as the prevalent genetic compositions of the virus have changed in many different ways over time. As described previously, Table 2 shows the demographic distribution for the patient metadata validation set obtained. By way of example, the B.1 lineage (D614G) is a very early lineage, first detected 1 January 2020 (https://cov-lineages.org/lineage_list.html, accessed on 14 November 2022) and, as Figure 4A shows, no longer highly prevalent by the mid-2020. In the validation data set, 73% of samples indicated a severe outcome for the B.1 variant, the ancestral genome with a D614G mutation. By contrast, only 60% of patient outcomes are severe for Alpha (B.1.1.7) sequences, and that drops to 42% for Delta (B.1.617.2) and further to 26% for AY.4 Delta sublineages. Although the average age is lower for Delta patients, that is insufficient to explain this drop, which is seen across sequences with higher ages as well. Overall, the results shown in Table 2 contradict repeated studies showing that Alpha resulted in more severe outcomes as evinced, for example, by increased hospitalizations and ICU admissions, and that Delta was yet more severe [78,79,80,81,82,83,84].

The data, however, cannot account for changes in how COVID-19 is treated and can be prevented. In particular, it is not possible to directly control GISAID data for vaccination, as there is still very limited information about vaccination for samples in GISAID metadata (at least as of November 2021). The decline of mean case severity in early 2020 is similar to findings of a Canadian study which reported a decrease in case fatality rate (CFR) between the first and second waves, occurring prior to any vaccination, even when controlling for the increased incidence in younger individuals and greater testing rates [85]. The latter decline may be accounted for by better understanding of how to treat COVID-19, and, later in 2020, the emergence of monoclonal antibody therapies. The accelerated decline in case severity starting in early 2021 is likely attributable to vaccination, particularly of older populations, which would also be consistent with the lower mean ages of Delta and its sublineages as indicated in Table 2. Countries in which vaccines were widely available among the elderly in early 2021 and general population through 2021 are highly overrepresented in GISAID sequence data: of all sequences in GISAID through the October 2021 cutoff, 55% were in Europe and 34% were from North America. The overrepresentation is less acute in the subset with patient metadata, but still 69% of the GISAID sequences with metadata for patient status were from Europe and North America combined.

Accordingly, sample collection date is used a demographic variable in training the neural network, along with age and gender. Date, then, serves as an indirect proxy for dynamic changes in diagnosis, treatment, and vaccination in the course of the pandemic. As shown in Figure 1, these variables are fed into the neural network model at the *N_H_*-dimensional densely connected layer. A sample’s demographic variables are thus embedded along with the spike protein sequence in the *N_H_*-dimensional embedding vectors obtained from that layer. Table 3 shows the classification metrics of the Transformer-based neural network model and XGBoost on the validation data set. The training and validation sets are constructed to avoid sequence overlaps, as detailed in the Methods section. The results of the neural network model are compared to XGBoost, as it is a decision tree-based ensemble learning approach that has proven to be useful in biological sequence machine learning applications as it is (1) fast, (2) highly effective at classification, (3) robust to missing values (which frequently arise in GISAID data due to missing or ambiguous amino acid codes), and (4) can provide a ranking of feature importance, which are used to validate the interpretability of the neural network model below [86,87]. For XGBoost modeling, the sequence is one-hot encoded such that each amino acid found at each sequence position in the data, with "NaN" used to represent missing values, i.e., where there is padding “*” or an amibiguous amino acid “X”.

Overall, as Table 3 shows, both the neural network model and XGBoost are able to correctly predict a substantial majority of both the mild and severe cases. XGBoost does outperform the neural network model when including demographic variables and sequence information together. The XGBoost model performs at best equally well when not including demographic and considering sequence alone. The difference in performance can be accounted for by XGBoost’s better ability to handle demographic variables, which is consistent with the superior ability of gradient boosting methods to handle tabular data as compared to deep neural networks [88]. However, a crucial drawback of the XGBoost model is that, by contrast to the neural network model, the XGBoost model as implemented here cannot be generalized. The XGBoost model utilizes one-hot encoding of amino acid variations at different sequence positions, and as a result could not model novel sequence rearrangements and mutations such as those found in emerging Omicron variants. The one-hot encoded sequence representation could theoretically include all possible amino acid positions, in which case the feature vector would have needed to include very large sparse feature vectors for a large number of samples resulting in very slow training and poor algorithm convergence. However, more importantly, because there is no sequential information in a XGBoost model, it cannot make reproducible predictions for sequence changes that it has not seen before, and as such proved unable to make reproducible predictions for Omicron. By contrast, the Transformer-based neural network model proved able to generalize to consistent predictions that were qualitatively accurate for Omicron, as discussed below. We further note that the performance of deep learning is markedly worse on the minority class (Mild cases) suggesting that improved class balancing, such as methods for oversampling data, may result in improved deep learning performance.

The combined sequence and demographic model can also be qualitatively validated. Figure 5D shows predictions made by training the Transformer-based neural network model using sequence as well as age, gender, and sample collection date.The trained model was simulated for dates starting in March 2020, well before either Alpha (early 2021) and then later Delta (mid-2021) were prevalent and their sequences had emerged (September 2020 and December 2020 respectively) [89,90]. As expected, the model predicts that Alpha and Delta cases would have been more severe had they occurred earlier in the pandemic. Moreover, the model predicts that, overall, Delta cases would be more severe than Alpha, in agreement with the above-cited clinical and epidemiological literature. In sum, once sample collection date is incorporated in the model, the resulting predictions can be validated both quantitatively and qualitatively.

### 3.3. Interpreting the Disease Severity Model by Visualizing Embeddings and Attention

**Figure 6:**
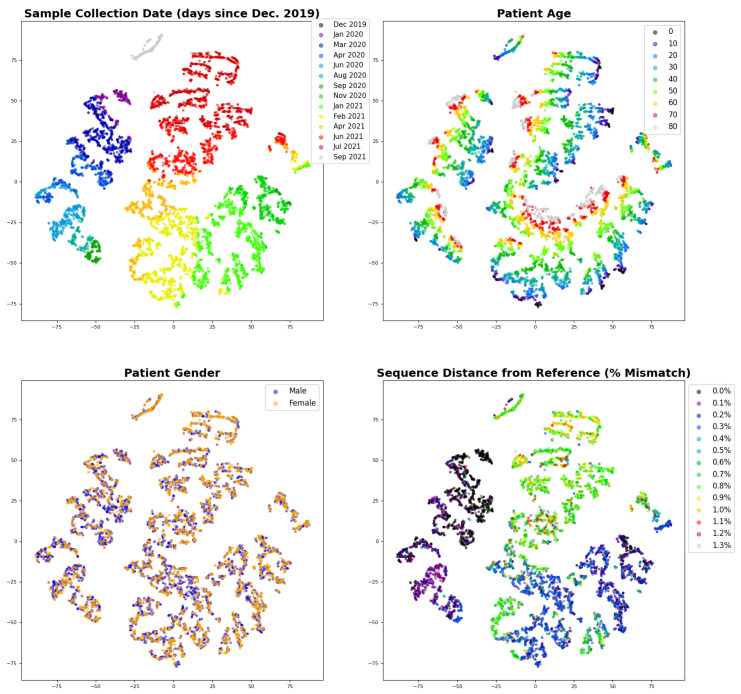
Sequence embeddings obtained from the trained neural network model and visualized based on a 2-component t-SNE (perplexity of 30). The plots all show the same t-SNE visualization of a random sampling of 150 samples each of the 45 most prevalent lineages in order to achieve sequence and sample date diversity. Clockwise starting with the upper left, the graphs show the t-SNE distribution labeled by sample collection date, patient age, sequence distance, and gender respectively. Variables are binned as indicated in the legend. Sequence distance is the number of mismatches between the sequence of the sample to the reference protein sequence divided by the reference protein sequence length, disregarding any padding or ambiguous amino acids in the sample sequence. The embeddings are heavily influenced by variant emergence and thus time, in addition to age. Gender has little effect.

To visualize sequence embeddings, random equal samples are obtained from the 45 most prevalent lineages. Sampling allows the visualization to be more tractable while retaining the structure of the data, i.e., to avoid having lineages that have many more sequences in the database, particularly those from Alpha and Delta lineages, swamp the signal from other sequences. Figure 6 shows how the different input variables, sequence, age, gender, and sample collection date, influence the embedded structure of the validation data set. The effect of sequence on a sample’s embedding is visualized by quantifying the distance between the sample’s spike sequence and the spike reference sequence, which is the consensus of ancestral genomes. Sequence distance is measured by the mismatch frequency obtained by dividing by length of the spike reference sequence as a percent mismatch.

It is expected that the t-SNE plots of the embeddings in Figure 6 should reflect how the model performs its ultimate regression. The *N_H_*-dimensional embedding layer is the layer immediately before the final sigmoid-activated node that outputs the class probability (i.e., probability that a sample is from a severe case) which is rounded to the mild/severe (0/1) prediction. Therefore, at the *N_H_*-dimensional layer, embedded samples will be separated in the embedding space in such a manner that they can be most accurately classified based on the trained model parameters. The t-SNE plots should thus accentuate similarities and distances, the visualization should reflect the separation and clustering of the embeddings according to how they will be classified.

Figure 6 shows, as expected, consistent patterns within the relative positions of sample embeddings according to age, sample date, and sequence distance. The exception is gender, which fails to show separation between male and female samples. The lack of a pattern for gender in Figure 6 is consistent with Figure 5B, which shows that the effect of gender on disease severity is not as significant as that for age or sample date. Severe cases account for 58% of samples from male patients, as opposed to 53% from female patients. (As noted above, the overall likelihood of severe cases in the GISAID-sourced data set is much higher than would be expected in the general population.) By contrast, age and sample date do play an important role. As Figure 6 shows, the patterns of age and sample date are orthogonal to each other. The relationship between age and sample date patterns is consistent with Figure 5A,C, which show that as age increases, severity increases, but as sample date increases, severity decreases. Figure 6 also shows that sequence is playing a role in the model prediction. The effect of sequence variation is represented by categorizing samples according to their degree of difference (distance) from the reference sequence that was used for alignment, as described in the Methods. The sequence distance is measured by mismatch frequency normalized by the length of the reference sequence (1273 amino acids). Figure 6 shows a pattern of separation and clustering among samples by sequence distance that is clockwise similar to sample date as the two will be correlated as discussed above. In sum, Figure 6 shows a visualization how the neural network model utilizes the input variables to train the model.

We can also gain insight into the model through attention. To demonstrate that the attention of a sequence position in the model can correspond to the importance of that position to classification, the attention values found at different positions in the spike protein sequence are compared to XGBoost feature importance scores. Trained XGBoost models can provide a prediction of feature importance by interpreting scoresbased on characteristics of boosted tree models: for example, the number of times a feature is used to split trees or the gain in score (i.e., difference from objective) obtained by splitting trees based on a feature [91,92].

Table 4 shows a comparison of the XGBoost feature score (gain) and attention score for the highest attention sequence positions. The ranking of the XGBoost feature score is related to the rank of the highest attention sequence features. The highest-scoring features in the XGBoost model, however, were age, sample date, and gender. This is likely because XGBoost uses a greedy approach to branch decision trees in variables that will most rapidly lead to the optimal classification [64]. Therefore, it is expected that XGBoost will consider gender as an important variable as it varies consistently in the training samples, while samples may have very similar or even identical sequences. By contrast, as Figure 6 shows, it is less important to the Transformer-based neural network model. The XGBoost feature scores in Table 4 are likewise much more skewed towards a smaller number of variants than the attention scores from the neural network model. Despite these differences, as Table 4 indicates, the 10 most important sequence features per XGBoost were found among the 22 highest mean attention positions, showing a concordance between the attention and XGBoost scores.

**Figure 7:**
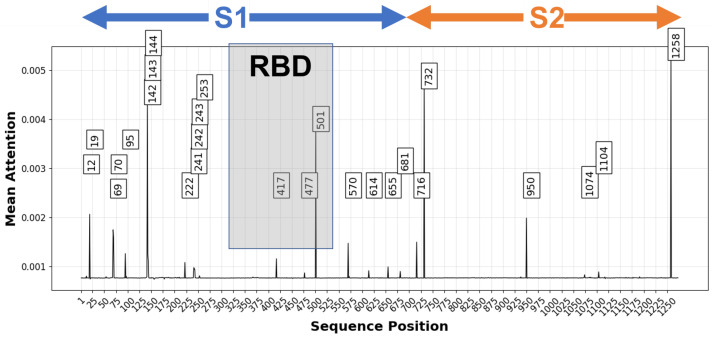
Mean attention at each spike protein sequence position of validation samples. Labeled positions have attention scores above 0.0008. The graph approximate locations for the S1 and S2 subunits, which is cleaved at a furin cleavage site (positions 680-689) by host proteases leading to membrane fusion and host cell entry [4]. The receptor binding domain (RBD) (391-541) is also shown [94]. Many high attention sites are clustered to the N-terminal domain (NTD) and C-terminal domain (CTD) of the S1 subunit.

Figure 7 shows a the mean attention obtained from running the validation samples through the model plotted across the spike protein sequence. Many high attention positions coincide with sites previously identified as potentially significant for virulence, infectivity, and/or immune evasion. For example, at the third-highest position, protein structure analysis suggests that G142D and deletions at 143–144, present in some Delta and Delta sub-lineage sequences, may be linked to higher infectivity and immune evasion [93]. The highly ranked sites also include locations known to be significant for differentiating SARS-CoV-2 variants, such as positions 69 (corresponding to the 68–69 deletion found in B.1.1.7/Alpha), 501 (where the N501Y mutation occurs, which is characteristic of B.1.1.7/Alpha and found in some Delta variants), and 681 (P681R and P681H found in Delta and Alpha respectively) [94]. By contrast, another site with frequent mutations, site 484, where E484K, E484Q, and E484A mutations associated with antibody escape have been found [71], does not have high attention. The low attention at position 484 in the disease severity model may suggest that the site does not have a significant role in determining virulence rather than antibody evasion [26,70,72,73,74].

The site with the highest mean attention, position 1258, however, has not been as well characterized. The most common mutation found in the training and validation data at that site was E1258D, although E1258H was also found. E1258Q has been observed before as well, and the site has been linked to coincident mutations that may result in a spike protein missing its terminal region [95]. It appears that these spike mutants would cause the spike protein to accumulate in the plasma membrane, which would result in syncytia, large multinuclear cellular masses, which may result in heightened virulence [95]. Such truncated spike proteins are not observed in the patient data set. However, E1258D is associated with much higher disease severity. Among the 2.6% of samples with an E1258D mutation, 99% were categorized as severe cases. (The model correctly predicted that 92% of them would be severe.) Another report indicated that E1258D had been found in Delta sublineages, but without additional analysis of its potential impact on patient outcomes or transmissibility [96].

Importantly, E1258D is not lineage specific: 38.7% of the E1258 in the patient data set were from B.1.1.519, 18.1% were P.1 (Gamma), 14.8% B.1.1.7 (Alpha), and 11.9% were B.1.617.2 (Delta) and Delta sublineages (primarily AY.3 and AY.20). As a result, the conventional way of analyzing virulence by studying specific lineages would not pick up on a difference that is due to the presence of E1258D. This demonstrates how sequence modeling can be a crucial complement to phylogenetic and other lineage-based classifications. E1258D is an example of a mutation that can be adopted, and sometimes can revert, within multiple clades, and different times, and along with different combinations of other mutations. While it is possible that the E1258D mutation’s apparent importance from the deep learning analysis of the GISAID patient data set may be an artifact of the limited data analyzed, the strong signal suggests that it may warrant further investigation.

### 3.4. Predicting Omicron Disease Severity

**Figure 8:**
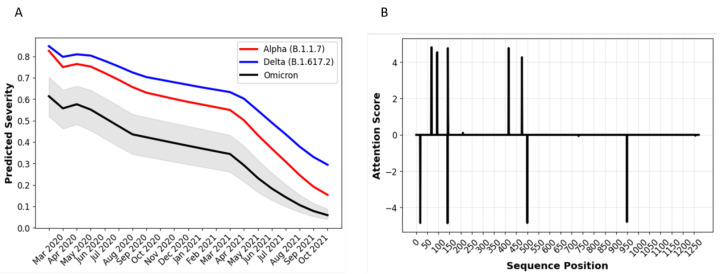
(**A**) Model prediction of the average time-dependent disease severity, assuming a 50-year-old male patient, for samples with known sequences from Beta, Delta, and Omicron lineages. Omicron is predicted to have, on average, a 35–40% reduced risk of severe disease as compared to Delta, and to have reduced risk relative to Beta as well. (**B**) Differential attention plot showing the spike protein sequence positions that have the greatest difference in attention score between most commonly found Delta and Omicron sequences. Sites with a greatest positive attention difference are 69–70 (where Omicron has a deletion also present in Alpha but not Delta), 95 (the most common Omicron sequence type, shown here, lacks Delta’s T95I mutation), 143–144 (Omicron has a deletion), 211 (Omicron has a deletion), 417 (Delta lacks the K417N found in many but not all Delta lineages), and 477 (Omicron lacks S477N). Sites with the greatest negative attention difference are 19 (T19R found in some but not all Delta sequences), 142 (Omicron lacks Delta’s D142G), 501 (N501Y also found in Alpha) and 950 (Omicron lacks N950D).

As discussed above and shown in Figure 4C, since Omicron emerged at a later date in the pandemic than other variants, it would likely prove be less severe in terms of real world infections than if the same variant had emerged earlier in the pandemic—simply because of improved vaccination, increased immunity due to previously acquired infections, and improved therapeutics. Accurately predicting the impact of Omicron or any other newly emerging variants thus requires controlling for age and date, as shown Figure 5D, to compare Omicron’s relative severity to other variants. Figure 8A shows how the mean predicted disease severity of Omicron sequences would vary over time through the pandemic. Predicted disease severity varies less between sequences than predicted immune evasion; however, there is still some variability as Figure 8A shows. The maximum standard deviation in the probability of a severe case was found to be less than 0.01 for all time periods, and the largest peak to peak variation is ± 15%. As Figure 8A illustrates, even the worst-case scenario which would still result in a significantly lower severity than Delta, accounting for the variability of predictions between Delta sequences.

Figure 8B shows a differential attention plot, which indicates the spike protein sequence locations that have the greatest difference in attention score between the most prevalent Delta and Omicron sequences. The model found mutations at these sites to be most relevant in predicting that Omicron would likely be less severe than Delta (controlling for patient age, sex, and case date). Further study is required to determine the potential effect of the spike protein sites and mutations identified in Figure 8B on biological mechanisms that could affect infectivity or cell-cell fusion in ways that would impact the risk of severe outcomes.

## 4. Discussion

The reduced severity of Omicron as compared to Delta is consistent with early laboratory evidence that there is a reduction in lower lung infectivity, deficient cell entry, and syncytium formation likely due to diminished mediation of plasma membrane fusion by the modified spike protein [97,98]. Omicron has also been found to be less infective in the lung and cause less severe disease in a Syrian hamster model [99,100]. Figure 8A further shows a reduction in predicted Omicron severity as compared to the Beta variant that had been prevalent in Southern Africa pre-Delta and which also has a high degree of immune evasion. The decrease in severe disease is also consistent with preliminary clinical and epidemiological evidence that had emerged by the time of this manuscript’s submission, suggesting that patients had less severe disease in South Africa [101]. Reduced severe disease in South Africa may be due to a particularly high degree of seroprevalence in South Africa as a result of prior infection and vaccination of the vulnerable population. However, early data from the United Kingdom also suggest a 20–25% reduced level of hospitalization of any kind and 40–45% reduction in hospitalizations of greater than one day [102]. A recent report of medical records in the United States showed that as Omicron replaced Delta as the predominant variant, there was a greater than 50% reduction in 3-day risks of various indicators of severity, including hospitalization, ICU admission, and mechanical ventilation [103]. The model’s prediction that Omicron may be more severe than other VOC has also been supported by early reports. It is difficult to compare clinical and epidemiological evidence of severity for variants earlier in time, because of the effect of vaccination, previous infections, and improved therapies. However, the prediction of reduced severity is consistent with the preliminary data showing that Omicron may have deficient cell entry and less induction of cell-cell fusion as compared to wild type (ancestral genome) as well as Delta [97]. Free energy calculations also suggest that the Omicron spike protein has deficient host cell receptor binding as compared to wild type or Delta [104].

The results shown here, a key impediment to deep learning is the risk of overfitting due to insufficient training data. When models overfit, they can only explain training data—and unable to make predictions on sequences or patients that the model has not seen before. This is a particular challenge in biomedical problems, where clinical and experimental data demand time, specialized expertise, effort, and financial cost [105]. Even so, the results shown in this paper demonstrate that the model can predict that Omicron has substantially less risk of causing severe disease than the Delta variant. This prediction has proved to be consistent with empirical reports. The ability of the model to make validated predictions for Omicron, despite Omicron’s novelty and distance from previously observed SARS-CoV-2 sequences shows that the deep neural network framework is generalizable. This study therefore provide the proof of concept for a computational modeling framework that can provide predictive insight on the properties of potential emergent SARS-CoV-2 variants—before empirical data for that variant become available. Future work will build on the approach used for modeling the effect of vaccination using sample collection date as a proxy with more sophisticated models of the effect of vaccination on COVID-19 infection dynamics, e.g., [106].

There are limitations in the demographic information that can be included given that the sequence data on GISAID does not have additional information. As a result, the model cannot account for other important factors that increase the risk of severe COVID-19, including comorbidities and racial disparities [107]. Also, patient metadata may be snapshots in time. For example, a mild case may evolve into a severe case without updating its annotation. This study also does not consider the geographic origin of cases. The severity of cases may be related to different countries and regions’ enforcement of non-pharmaceutical interventions (NPI), population mixing (and differential impact of lockdowns and other restrictions), levels of infection, and differences in hospital capacity [108,109,110]. However, there is a strong confounding effect between particular sequence variations, even within lineages, and countries (or even regions within large countries, such as the United States) [111]. As a result, the relationship between sequence and severity will in fact reflect regional-specific factors to some extent, particularly for sequences that do not become widespread in multiple regions. However, given the small fraction of sequences for which patient metadata are available, there are too few sequenced samples in individual countries or regions to enable machine learning based on geographic variables. Future work may allow for the inference of pandemic characteristics based on dynamic disease models, such as the approach of epidemiological modeling utilizing fractional dynamics employed in Pakistan [112].

Finally, while there is an unprecedented amount of sequencing data available for SARS-CoV-2, the tasks demonstrated here in large part only used thousands of the millions of available samples. This was due to the lack of metadata beyond geographic origin and simple demographic variables for the vast majority of sequences. An important objective of sequencing work should be to collect and curate standardized information about the sample and sequence techniques [113]. However, the fact that the model presented here can provide insight to SARS-CoV-2 even with limited data sets shows the potential for the deep modeling approach shown here to be extended to other biological problems.

## 5. Conclusions

This paper shows that a deep sequence model that incorporates a multi-attention Transformer as well as sequence-wide self-attention and embedding layers for multiscale interpretation can successfully perform classification and regression tasks on SARS-CoV-2 spike protein sequence data. The results in this paper demonstrate that the modeling approach shown here can identify *attention* values that correspond to sequence positions of interest and *embeddings* that can be visualized to show relationships between sequences. Modeling the relationship between disease severity and sequencing variants sets requires accounting for not only patient age but also sample collection date, particularly due to time-dependent increases in vaccination rates. Accounting for these differences by combining demographic variables with sequence input to the deep learning model allows for the prediction of patient severity from GISAID data at nearly 70% accuracy. The model can also qualitatively predict that Delta is more severe than Alpha when controlling for time as a proxy for vaccination and improved therapeutics. Furthermore, the model predicts that Omicron is less severe than both Delta and Alpha, accounting for the confounder of yet increased levels of vaccination and immunity due to prior infection, in agreement with experimental evidence.

## Figures and Tables

**Table 1:** **Classifications based on metadata entries.** Metadata entries are translated to a Status, following the definitions in Supplemental Table S1, and then assigned to Category, which is in turn classified as Mild, Severe, or Unknown.

Category	Status (Based on Table S1)	Classification
Alive	Alive	Unknown
Asymptomatic	Asymptomatic, Asymptomatic/Hospitalized, Asymptomatic/Mild, Asymptomatic/Mild, Paucisymptomatic, Paucisymptomatic/Reinfection	Mild
Dead	Dead, Dead/Vaccinated	Severe
Hospitalized	Hospitalized, Hospitalized/Stable, Hospitalized/Symptomatic	Severe
Mild	Ambulatory, Ambulatory/Under treatment, Home, Hospitalized/Mild, House Assistance, Mild, Mild/Reinfection, Mild/Moderate, Not hospitalized, Not severe, Outpatient, Vaccinated/Mild	Mild
Moderate	Hospitalized/Moderate, Hospitalized/Not Serious, Moderate, Moderate/Reinfection, Moderate/Severe, Not critically hospitalized, Pneumonia	Mild
Released	Released	Severe
Screening	Screening	Mild
Symptomatic	Acute infection, Bronchitis, Chronic infection, Flu-like, Stable, Symptomatic, Symptomatic/Reinfection	Uknown
Severe	Casualty, Critical, Hospitalized/Serious, Hospitalized/Serious, ICU, Serious, Severe, Severe/Hospitalized	Severe
Unknown	Active, Not vaccinated, Recovered, Reinfection, Unknown, Unknown/Vaccinated, Vaccinated	Unknown

**Table 2:** **Lineage-specific statistics of GISAID samples with metadata.** Mean age, percentage of samples which are from patients with severe COVID-19 disease, percentage of samples from male populations, and the mean sample collection date of samples in the GISAID patient metadata validation data set from the lineages that appeared at least 400 times (i.e., frequency > 400). Mean sample collection date is defined in terms of days since 1 December 2019. The most recent lineages (highest sample date) trend to lower severity.

Lineage	Mean Age	% Severe	% Male	Mean Date	Frequency
**AY.4**	39.4	26%	53%	631	817
**B.1**	49.8	73%	42%	314	994
**B.1.1**	50.3	70%	49%	314	696
**B.1.1.519**	49.9	68%	47%	485	1583
**B.1.1.7**	48.3	60%	50%	509	3298
**B.1.351**	45.7	52%	50%	481	849
**B.1.617.2**	40.0	42%	48%	604	3901
**P.1**	48.3	64%	48%	548	877

**Table 3:** **Classification metrics for the patient metadata validation set for the neural network model of Figure 1 and XGBoost model trained on individual samples.** Prediction results shown are based on training on age, gender, and date as the only independent variables, sequence alone, and the sequence combined with other variables. There are 8948 and 12,965 samples of Mild and Severe patient outcomes respectively. Because of the imbalanced classes, the class-specific metrics are shown here in addition to overall accuracy. The value on the left of the forward slash corresponds to the Mild class, and the value on the right to the Severe class. Classification accuracy of the Transformer-based neural network model approaches 70% and is comparable to XGBoost.

	Precision	Recall	*F*_1_ Score	Accuracy
**XGBoost**				
No Sequence	0.56/0.72	0.65/0.64	0.60/0.68	0.64
Sequence Only	0.57/0.74	0.66/0.65	0.61/0.69	0.65
Sequence + Age, Gender, Date	0.64/0.77	0.67/0.74	0.66/0.76	0.71
**Transformer**				
No Sequence	0.53/0.75	0.72/0.56	0.61/0.64	0.63
Sequence Only	0.58/0.69	0.52/0.74	0.55/0.72	0.65
Sequence + Age, Gender, Date	0.59/0.76	0.70/0.66	0.64/0.71	0.69

**Table 4:** **Amino acid positions with the highest mean attention and XGBoost scores.** Attention rank and scores are compared to the XGBoost feature importance ranking and scores (obtained from the gain measure of feature importance). This table includes the 10 highest-scoring features identified by XGBoost, which appear within the 22 highest attention values.

Attention	Amino Acid	Mean Attention	XGBoost	XGBoost
Rank	Position	Score	Rank	Score
**1**	1258	5.42	**73**	163
**2**	732	5.1	**10**	2090
**3**	142	4.96	**1**	6421
**4**	501	3.79	**8**	2272
**5**	69	2.14	**5**	2813
**6**	70	2.13	**31**	573
**7**	716	2.08	**25**	756
**8**	570	2.06	**27**	709
**9**	19	1.99	**26**	720
**10**	950	1.92	**17**	1352
**11**	144	1.34	**6**	2306
**12**	95	1.25	**2**	4698
**13**	222	1.07	**4**	3474
**14**	417	1.06	**24**	757
**15**	655	1.01	**62**	225
**16**	614	0.94	**7**	2288
**17**	1104	0.91	**9**	2240
**18**	241	0.87	**134**	78
**19**	243	0.86	**335**	7
**20**	242	0.86	**250**	23
**21**	477	0.86	**14**	1488
**22**	681	0.84	**3**	3559
**23**	1074	0.83	**22**	962
**24**	97	0.81	**18**	1168

## Data Availability

The datasets analyzed for this study were downloaded from GISAID EpiCoV database pursuant to the GISAID terms of use and from NCBI GenBank. The sequences obtained from GISAID are available for download to users who register with GISAID at the website http://wwww.gisaid.org, accessed on 14 November 2022. The list of GISAID accession numbers used for this paper and data acknowledgments are provided collectively by the permanent GISAID sequence identifier EPI_SET_20220105ms, and the accession numbers and complete set of data acknowledgments are available at https://epicov.org/epi3/epi_set/EPI_SET_20220105ms, accessed on 14 November 2022. The code used for pre-processing and analysis in this paper has been deposited to and made publicly available from the authors’ GitHub repository, https://github.com/bahrad/Covid, accessed on 14 November 2022.

## Supplementary Materials

The following supporting information can be downloaded at: https://www.mdpi.com/article/10.3390/biology11121786/s1. Table S1. GISAID Patient Status Metadata Encodings. Mapping scheme used for raw patient status metadata. The mapping is to a patient status which is then mapped to a category and classification according to Table 1 as detailed in the Methods section.

## Abbreviations

The following abbreviations are used in this manuscript:

BERT	Bidirectional encoder representations from transformers
Bi-LSTM	Bidirectional long short term memory
CNN	Convolutional neural network
CTD	C-terminal domain
NLP	Natural language processing
NTD	N-terminal domain
RBD	Receptor binding domain
RNN	Recurrent neural network
SARS-CoV-2	Severe acute respiratory syndrome coronavirus 2
t-SNE	t-distributed stochastic neighbor embedding
VOC	Variant Of Concern
