# Supplementary material for: Interpretable and Predictive Deep Neural Network Modeling of the SARS-CoV-2 Spike Protein Sequence to Predict COVID-19 Disease Severity
Source: Biology (Basel). 2022 Dec 8;11(12):1786. doi: 10.3390/biology11121786 (PMC9774807; doi:10.3390/biology11121786)
Supplement: Supplementary file 1 [file biology-11-01786-s001.zip › SuppTableS1.pdf]

Original	Mapped Value
18	Unknown
28	Unknown
37	Unknown
38	Unknown
42	Unknown
43	Unknown
44	Unknown
45	Unknown
51	Unknown
54	Unknown
55	Unknown
58	Unknown
59	Unknown
61	Unknown
67	Unknown
68	Unknown
71	Unknown
103	Unknown
Active	Active
Active case	Active
Acute bronchitis	Acute infection
Acute upper respiratory infection, unspecified	Acute infection
Acute upper respiratory infections	Acute infection
Acute upper respiratory tract infection, unspecified	Acute infection
acute viral respiratory infection	Acute infection
Admitted for 6 Days in Bharatpur Hospital	Hospitalized
Airport surveillance	Screening
Alive	Alive
Ambulant	Ambulatory
Ambulatoriale	Ambulatory
Ambulatory	Ambulatory
AMBULATORY - UNDER TREATMENT	Ambulatory / Under treatment
Ambulatory symptomatic	Mild
Ambulatory treatment	Ambulatory
Amublant	Ambulatory
Animal showing generalized sickness	Unknown
ARV clinic	Unknown
Asympomatic	Asymptomatic
asymptomatic	Asmptomatic
Asymptomatic - Ambulatory	Asymptomatic
Asymptomatic ambulatory	Asymptomatic
Asymptomatic and Ambulatory	Asymptomatic
Asymptomatic, identified as positive during preoperation investigation	Asymptomatic
Asymptomatic/Mild	Asymptomatic/Mild
Asymptomatic/Released	Asymptomatic
Asymptomatyc	Asymptomatic
Asymtomatic	Asymptomatic
Aymptomatic	Asymptomatic
B34.2 Coronavirus infection, unspecified site	Unknown
Blank	Unknown
Bronchitis	Bronchitis
Bronchoalveolar lavage	Unknown
Casualty	Casualty
Chronic dialysis	Unknown
Clinic	Unknown
Clinical signs	Symptomatic
Cluster	Unknown
Community screening	Screening
Community Surveillance	Screening
Confirmed	Unknown
Confirmed SAD	Unknown
Confirmed SAD-SAD	Unknown
Confirmed SCTE	Unknown
Conjunctivitis	Mild
Contact	Screening
Contact and possible infection with other infectious diseases	Screening
Contact surveillance	Screening

Contact with and exposure to other communicable diseases	Screening
Contact-SAD	Screening
Contained	Unknown
Coronavirus infection	Unknown
Cough	Mild
COVID-19	Unknown
Critical	Critical
critical care admitted	Severe
Culled for pelting	Unknown
Cured	Recovered
DAMA	Unknown
Dead	Dead
dead or alive, hospitalised or release	Unknown
Dead, hospitalized	Dead
Death	Dead
Decease	Dead
Deceased	Dead
Deceased (BID)	Dead
Deceased (Brought in Dead)	Dead
Decreased	Dead
Demise	Dead
Demised	Dead
Demised patient	Dead
Demised patient (Vaccinated first dose)	Dead
Desceased	Dead
Deseased	Dead
Died	Dead
Discharged	Released
Discharged after recovery	Released
Diseased	Symptomatic
Domestic infection	Unknown
EHPAD	Unknown
EHPAD_IRA	Unknown
Emergency care	Hospitalized
Encounter for general adult medical examination	Screening
Encounter for general adult medical examination (Z.00)	Screening
Encounter for observation for other suspected diseases and conditions	Symptomatic
Encounter for other specified special examinations (Z01.8)	Unknown
Epidemiology Study	Screening
Etude	Unknown
Exitus	Dead
Expired	Dead
Facility quarantine	Screening
Family medical ward	Hospitalized
Fatal	Dead
Female	Unknown
Fever	Mild
Flu-like symptoms	Flu-like
General clinic	Unknown
General Medical Screening	Screening
HCW - Mild Covid	Mild
Headache	Mild
Headache, Nasal Congestions and generalised body ache	Mild
Healthcare worker	Unknown
Healthworker	Unknown
Helen Joseph ward	Unknown
High temperature	Mild
Home	Home
Home Isolation	Home
Hopsitalized	Hospitalized
Hopspitalized	Hospitalized
Hospatilized	Hospitalized
Hospetalized	Hospitalized
Hospidalized	Hospitalized
Hospital discharge	Released
Hospitalised	Hospitalized
Hospitalised oncology urology	Hospitalized
Hospitalised-live	Hospitalized
Hospitalised, Reinfection	Hospitalized

HOSPITALIZADO	Hospitalized
hospitalized	Hospitalized
Hospitalized - Non-Serious Case	Hospitalized / Not Serious
HOSPITALIZED - SERIOUS CASE	Hospitalized / Serious
Hospitalized -ICU-critical	Severe
Hospitalized (Critical)	Hospitalized / Serious
Hospitalized (Deceased)	Dead
Hospitalized (ICU)	Severe
Hospitalized (Intensive care unit and Released)	Severe
Hospitalized (Intensive care unit)	Severe
Hospitalized (Live)	Hospitalized
Hospitalized (Mild)	Hospitalized / Mild
Hospitalized (Moderate)	Hospitalized / Moderate
Hospitalized (possible MIS-C)	Hospitalized / Serious
Hospitalized (Released)	Released
Hospitalized (Severe)	Severe
Hospitalized (Stable)	Hospitalized / Stable
Hospitalized / ICU	Severe
Hospitalized / Rawat Inap	Hospitalized
Hospitalized Asymptomatic	Asymptomatic / Hospitalized
Hospitalized in intensive care	Severe
Hospitalized Symptomatic	Hospitalized / Symptomatic
Hospitalized, Asymptomatic, Alive	Asymptomatic / Hospitalized
Hospitalized, Deceased	Dead
Hospitalized, Discharged, Alive	Released
Hospitalized, ICU	Severe
Hospitalized, ICU, deseased	Dead
Hospitalized, ICU, Died	Dead
Hospitalized, Live	Hospitalized
Hospitalized, Live.	Hospitalized
Hospitalized, no clinical signs	Asymptomatic / Hospitalized
Hospitalized, noninvasive ventilation	Hospitalized
Hospitalized, oxygenotherapy, diarrhea	Hospitalized
Hospitalized, Pregnant	Hospitalized
Hospitalized, referred to Nepalganj Teaching hospital, stayed for 10 da	Hospitalized
Hospitalized(ICU)	Severe
Hospitalizede	Hospitalized
Hospitalizes	Hospitalized
Hospitallized	Hospitalized
Hospitarized	Hospitalized
Hospitilized	Hospitalized
Hospitlalized	Hospitalized
Hosptalized	Hospitalized
House assistance, Symptomatic	House Assistance
Human	Unknown
ICD-10 Disease: B34.2 Coronavirus infection, unspecified	Unknown
ICD-10 Disease: B34.2 Coronavirus infection, unspecified site	Unknown
ICD-10 Disease: J00- Acute nasopharyngitis [common cold]	Mild
ICD-10 Disease: J00-J06 Acute upper respiratory infections	Acute infection
ICD-10 Disease: J02.9 Acute pharyngitis, unspecified	Acute infection
ICD-10 Disease: J06.8 Other acute upper respiratory infections of mult	Acute infection
ICD-10 Disease: J06.9 Acute upper respiratory infection, unspecified	Acute infection
ICD-10 Disease: J18.1 Lobar pneumonia, unspecified organism	Pneumonia
ICD-10 Disease: J18.9 Pneumonia, unspecified organism	Pneumonia
ICD-10 Disease: J40 Bronchitis, not specified as acute or chronic	Bronchitis
ICD-10 Disease: U07.1 COVID-19, virus identified	Unknown
ICD-10 Disease: U07.2 COVID-19, virus not identified	Unknown
ICD-10 Disease: U07.2 Virus not identified	Unknown
ICD-10 Disease: Z00.0 Encounter for general adult medical examinatio	Unknown
ICD-10 Disease: Z00.0 Encounter for general adult medical examinatio	Symptomatic
ICD-10 Disease: Z03.8 Encounter for observation for other suspected d	Symptomatic
ICD-10 Disease: Z03.8 Observation for other suspected diseases and co	Symptomatic
ICD-10 Disease: Z04 Encounter for examination and observation for ot	Unknown
ICD-10 Disease: Z04.8 Encounter for examination and observation for r	Unknown
ICD-10 Disease: Z10.8 Routine general health check-up of other define	Unknown
ICD-10 Disease: Z11.5 Encounter for screening for other viral diseases	Unknown
ICD-10 Disease: Z20.8 Contact with and exposure to other communica	Screening
ICD-10 Disease:J06.9 Acute upper respiratory infection, unspecified	Acute infection
ICD-10 Disease:Pneumonia	Pneumonia

ICD-10 Disease:Z00.00 Encounter for general adult medical examinati	Asymptomatic
ICU	Severe
ICU, Deceased	Dead
ICU, Live	ICU
ICU; Serious	ICU
Immunocompromised	Unknown
In-hospital	Hospitalized
In-patient	Hospitalized
Infection after vaccine/Live	Unknown / Vaccinated
Influenza Like Illness	Flu-like
Initially hospitalized, but now improved and discharged	Released
inknown	Unknown
Inpatient	Hospitalized
Intensive Care	Severe
Intensive Care Unit	ICU
Isolation	Unknown
J02.9 Acute pharyngitis, unspecified	Acute Infection
J03.9 Acute tonsillitis, unspecified	Acute Infection
J04.1 Acute tracheitis	Acute infection
J04.2 Acute laryngotracheitis	Acute Infection
J06 Acute upper respiratory infections of multiple and unspecified site	Acute Infection
J06.8 Other acute upper respiratory infections of multiple sites	Acute Infection
J06.9 Acute upper respiratory infection, unspecified	Acute Infection
J12.9 Viral pneumonia, unspecified	Pneumonia
J18 Bronchopneumonia, unspecified	Pneumonia
J18.8 Other pneumonia, organism unspecified	Pneumonia
J18.9 Pneumonia, unspecified	Pneumonia
J22. Unspecified acute lower respiratory infection	Acute infection
J41 Simple chronic bronchitis	Acute Infection
J81 Pulmonary oedema	Pneumonia
live	Alive
Live / Hidup	Alive
Live, acute respiratory infection	Acute infection
Live, ambulatory care	Ambulatory
Live, asymptomatic	Asymptomatic
Live, hospitalized	Hospitalized
Live, mild fever and weakness	Mild
Live, mild symptoms, at home	Mild
Live, not hospitalized	Not hospitalized
Live, Not Hospitalized.	Not hospitalized
Live, physical examination	Screening
Live, Recovered	Recovered
Live, symptomatic	Symptomatic
Live.	Alive
Live. Ambulatory	Ambulatory
Live. Not hospitalized	Not hospitalized
Lived	Alive
lLive	Alive
M62.9 Disorder of muscle, unspecified, high temperature	Symptomatic
Male	Unknown
Mild	Mild
Mild - not requiring supplementary oxygen	Mild
Mild / Contact exposure / Asymptomatic	Asymptomatic / Mild
Mild clinical signs	Mild
Mild clinical signs without hospitalization	Mild
Mild clinical signs without hospitalization. Diarrhoea	Mild
Mild clinical signs without hospitalization. Distorted ability to smell	Mild
Mild clinical signs without hospitalization. Distorted ability to smell ar	Mild
Mild clinical signs without hospitalization. Distorted ability to smell ar	Mild
Mild clinical signs without hospitalization. Distorted ability to smell.	Mild
Mild clinical signs without hospitalization. Distorted ability to taste	Mild
Mild Covid	Mild
Mild disease	Mild
Mild disease, full recovery, just rhinorrhea	Mild
Mild disease, persistent infection	Mild
Mild infection	Mild
Mild Sympthoms	Mild
Mild symptomatology	Mild
Mild Symptoms	Mild

Mild symptoms (fever, cardiovascular disorders)	Mild
Mild symptoms, not-hospitalized	Mild
Mild symtomatic case	Mild
Mild, at home.	Mild
Mild, live	Mild
mild, unhospitalized	Mild
Mild/Contact exposure/Asymptomatic	Asymptomatic / Mild
Mild/Live	Mild
Mild/Moderate	Mild / Moderate
Moderate	Moderate
Moderate / Outpatient	Moderate
Moderate, Live	Moderate
Moderate/Severe	Moderate / Severe
Multidisciplinary ICU	Severe
Nanopore	Unknown
Nasal swab	Unknown
Nasopharyngeal and oropharyngeal swab	Unknown
Nasopharyngeal and oropharyngeal swabs	Unknown
Nasopharyngeal swab	Unknown
Neurology ward, Hospitalised	Hospitalized
New York	Unknown
NICD outbreak investigation	Unknown
No clinical signs	Asymptomatic
No Data	Unknown
No Hospitalized	Not hospitalized
Non critical hospitalization	Not critically hospitalized
Non Hospitalized	Not hospitalized
Non Severe	Not severe
Non-hopsitalized	Not hospitalized
Non-hospitalized	Not hospitalized
Non-hospitalized, symptomps: cough	Mild
Non-hospitalized, symptomps: sore throat	Mild
Non-hospitalized, symptoms: runny nose	Mild
Not hospitalized	Not hospitalized
Not Quarantined	Unknown
Not vaccinated	Not vaccinated
Nursing Home	Unknown
Nursing house	Unknown
Nursing-home	Unknown
Obit - Death due to lack of oxygen in the health unit.	Dead
Oligosymptomatic	Mild
Oro-pharyngeal swab	Unknown
Oronpharyngeal swab	Unknown
Oropharyngeal swab	Unknown
Other	Unknown
Other acute upper respiratory infections of multiple sites	Acute infection
Other acute upper respiratory tract infections of multiple localization	Acute infection
Other unidentified viral infections	Unknown
ou	Mild
Oupatient	Not hospitalized
out-patient	Not hospitalized
Outaptient	Not hospitalized
Outgoing traveler	Unknown
Outpatient	Not hospitalized
OUTPATIENT - HOME MONITORING	Home
Outpatient Care, Alive	Not hospitalized
Outpatient mild disease	Mild
Outpatient-live	Not hospitalized
Outpatient, Mild disease	Mild
Outre mer	Unknown
Overseas inflow	Unknown
Paediatric casualty	Casualty
Paeds casualty	Casualty
Pauci-Symptomatic	Paucisymptomatic
Paucisymptomatic	Paucisymptomatic
Paucisymptomatyc	Paucisymptomatic
Paucisymtomatic	Paucisymptomatic
Paucisymtpmatic	Paucisymptomatic
Persistent infection	Chronic infection

Pneumonia	Pneumonia
Pneumonia (chest X-ray)	Pneumonia
Pneumonia (chest X-ray), not critical	Pneumonia
Pneumonia, unspecified organism	Pneumonia
Positive	Unknown
Post mortem	Dead
Quarantine	Unknown
Quarantine isolation	Unknown
Quarantined	Unknown
R05 Cough	Mild
R06.0 Dyspnoea, Orthopnoea, Shortness of breath	Symptomatic
R43.0 Anosmia	Mild
R50 Fever of other and unknown origin	Mild
R50.9 Fever, unspecified	Mild
R51 Headache	Mild
R53 Malaise and fatigue	Mild
random surveillance	Screening
Re-infection/moderate/live	Moderate / Reinfection
Rebased	Unknown
Recover	Recovered
Recovered	Recovered
Recovering	Recovered
Recovery	Recovered
Recovery 2021-04-18	Recovered
Referido	Unknown
Reinfected	Reinfection
Reinfection	Reinfection
Release	Released
Released	Released
Released / Lepas Rawat	Released
Released, Live	Released
Respiratory disease	Symptomatic
Retesting	Unknown
Routine	Screening
Routine general health check-up of other defined subpopulations	Screening
Sance Asymptomatic	Asymptomatic
Screen	Screening
Screened for travel	Screening
screening	Screening
Serious	Serious
Sever/hospitalised	Severe / Hospitalized
Severa	Severe
Severe	Severe
Severe (Hospitalizated)	Severe / Hospitalized
Severe / ICU	Severe / Hospitalized
Severe clinical signs	Severe
Severe infection	Severe
Severe symptoms with underlying asthma and hypertension	Severe
Severe, hospitalized	Severe
Severe, Live	Severe
Severo	Severe
Sick	Symptomatic
sore throat, sweating, T-norm	Mild
Stable	Stable
Stable in quarantine	Mild
Sudden death	Dead
Surveillance	Screening
Suspect	Unknown
Suspect-SAD	Unknown
Suspect/SAD/SAD	Unknown
Suspect/SAD/SAD/SAD	Unknown
Suspected	Unknown
Suspected Corona	Unknown
Suspected coronavirus infection	Unknown
Sympotomatic	Symptomatic
symptomatic	Symptomatic
Symptomatic mild disease	Mild
Symptomatic - Ambulatory	Ambulatory
Symptomatic - Hospitalized	Hospitalized

Symptomatic ambulatory	Ambulatory
Symptomatic and Ambulatory	Ambulatory
Symptomatic and Hospitalized	Hospitalized
Symptomatic COVID-19	Symptomatic
Symptomatic, Outpatient	Outpatient
Symptomatic/mild illness/Live	Mild
Symptomatic/Reinfection	Symptomatic / Reinfection
Symptoms indicative of upper respiratory infection	Symptomatic
Symptoms: fever, general disconfort, headache, cough, rhinorrhea.	Mild
Symptoms: fever, general disconfort, muscle or joint pain, headache, [illegible]	Mild
Symptoms: fever, general disconfort, muscle or joint pain, headache, [illegible]	Mild
Symptoms: fever, general disconfort, muscle or joint pain, headache, [illegible]	Mild
Symptoms: fever, headache, loss of smell.	Mild
Symptoms: general disconfort, muscle or joint pain, headache, cough,	Mild
Throat	Mild
Trauma	Symptomatic
U07.1 Coronavirus infection caused by the COVID-19 virus, the virus ha	Unknown
U07.1 COVID-19, virus identified	Unknown
U07.2 Coronavirus infection caused by COVID-19 virus, virus not identi	Unknown
U07.2 COVID-19, virus not identified	Unknown
UCI	Unknown
uknown	Unknown
Umknown	Unknown
Undetlying DM and Ischemic Heart Disease	Unknown
UNK	Unknown
unknkown	Unknown
unknonw	Unknown
unknow	Unknown
unknwon	Unknown
unkonown	Unknown
unkonwn	Unknown
unkown	Unknown
Vaccinated	Vaccinated
Vaccinated & Demise	Dead / Vaccinated
Vaccine escape	Vaccinated
Vaccine escape/ Live	Vaccinated
Vaccine escape/Live	Vaccinated
Vaccine escape/mild/Live	Vaccinated / Mild
Very Severe	Severe
Voyageur	Unknown
x	Unknown
Z00 General medical examination	Unknown
Z00.0 General medical examination	Unknown
Z01 Other special examinations and investigations of persons without	Unknown
Z01.7 Laboratory examination	Unknown
Z04.9 Examination and observation for unspecified reason	Unknown
Z11.5	Unknown
Z11.5 Special screening examination for other viral diseases	Unknown
Z13.9 Special screening examination, unspecified	Unknown
Z20.0 Contact with and exposure to intestinal infectious diseases	Screening
Z20.8 Contact with and exposure to other communicable diseases	Screening
